# Evaluating Medical and Surgical Treatments for Ectopic Pregnancy at a Tertiary Hospital in Dubai

**DOI:** 10.7759/cureus.69216

**Published:** 2024-09-11

**Authors:** Elham A Akbari

**Affiliations:** 1 Obstetrics and Gynecology, Latifa Hospital, Dubai, ARE

**Keywords:** laparoscopic surgeries, methotrexate ectopic, multiple-dose methotrexate protocol, ruptured ectopic pregnancy, single-dose methotrexate protocol, unruptured tubal ectopic pregnancy

## Abstract

Introduction: The current data about ectopic pregnancy in the UAE is limited, including the incidence, method of management, and its effectiveness. This study aimed to determine the frequency of medical and surgical management in the treatment of ectopic pregnancy and the efficacy of each modality used.

Method: Two hundred and nine patients were diagnosed with ectopic pregnancies in the years 2018 and 2019 in Latifa Hospital and were included in this study. The patients were treated with either intramuscular injection of methotrexate (single or two doses) or surgical management.

Results: Methotrexate was administered to 101 patients (48%). In 77 patients (76%), a single dose of methotrexate was administered, and in 24 patients (24%), two doses of methotrexate were administered. In the single-dose group, 75.3% (58 out of 77 patients) were successfully treated and completely recovered. While in the two-dose group, the success rate was 58.3% (14 out of 24 patients). On the other hand, among patients who underwent surgical management, the success rate was 97.2% (105 out of 108 patients). The difference in the success rates was statistically significant.

Conclusion: The results of the study showed that medical and surgical management were used almost equally in managing patients with ectopic pregnancy (48% vs. 52%, respectively). Furthermore, it was shown that single-dose methotrexate treatment was more successful than two doses. However, surgical management had the highest success rate among the three modalities.

## Introduction

Ectopic pregnancy (EP) is a complicated gynecologic emergency characterized by the implantation of a fertilized egg outside the uterine cavity, predominantly in the fallopian tube. The incidence is highest among women aged 25-34 years. Although the prevalence of ectopic pregnancy varies geographically, it accounts for almost 1-2% of all pregnancies worldwide, with an associated global mortality rate of 5-10% of all pregnancy-related deaths, despite advancements in medical technology. According to a study conducted in Saudi Arabia, there was no mortality case documented. Ectopic pregnancy requires prompt diagnosis and appropriate management to prevent serious complications such as hemorrhage and shock [1,2].

The exact mechanism by which fertilized egg implants are located outside the uterus is not completely understood; however, it is thought to involve a combination of genetic and environmental factors. Xia Wang et al. studied the alteration of the immune cells that play an important role in the processes of embryo implantation, stromal decidualization, and early placental development. It was found that alteration in the phenotype and the activity of immune cells in the Fallopian tubes contributed to the development of ectopic pregnancy [3].

Multiple factors can cause ectopic pregnancy. Pelvic inflammatory disease (PID) is one of the most common causes of ectopic pregnancy and accounts for approximately 50% of all cases. It is a bacterial infection that can cause inflammation and scarring of the fallopian tubes, leading to tubal damage and obstruction. Endometriosis, a condition in which the tissue lining the uterus grows outside the uterus, causes intra-abdominal and pelvic adhesions, which can increase the risk of ectopic pregnancy. A previous history of abdominal or pelvic surgery, such as cesarean section or tubal sterilization, can cause tubal damage, which can also lead to ectopic pregnancy. In addition, certain risk factors can increase the likelihood of ectopic pregnancy [4].

Women with a history of ectopic pregnancy have a 10% risk of recurrence in subsequent pregnancies. In contrast, those with a history of two or more ectopic pregnancies had a risk of> 25%. Sexually transmitted infections, particularly chlamydia and gonorrhea, increase the risk of ectopic pregnancy [5].

Some behavioral risk factors can also contribute to ectopic pregnancy, such as having multiple sexual partners and smoking. In patients who smoke, it is believed that it damages the fallopian tubes and impairs their ability to transport eggs, leading to extrauterine implantation of fertilized eggs. Finally, the use of assisted reproductive technologies, such as in vitro fertilization (IVF), can increase the risk of ectopic pregnancy compared with women who conceived naturally [5].

The diagnosis of ectopic pregnancy varies according to its clinical presentation. Early diagnosis is essential, as the clinical features may mimic those of other gynecological conditions. The most common symptoms include abdominal or pelvic pain and vaginal bleeding in the presence of a positive pregnancy test. The pain may be dull or sharp and radiate to the lower back or shoulder. Advanced cases of ectopic pregnancy can present with symptoms such as syncope, severe abdominal pain, or hemorrhagic shock, which are signs of a ruptured ectopic pregnancy that require immediate medical attention. These symptoms can occur as early as 4-5 weeks of pregnancy [5].

A combination of clinical examination, ultrasound imaging, and serum β-hCG levels is typically used to diagnose ectopic pregnancy [5]. After obtaining a detailed history, a pelvic examination was conducted, which revealed an enlarged or tender ovary, a palpable mass, or free fluid in the cul-de-sac. Ultrasound imaging is the most reliable method for diagnosing ectopic pregnancy, with transvaginal ultrasound being the preferred modality owing to its high sensitivity and specificity [6]. Ultrasound can detect the presence of an ectopic pregnancy as well as any associated complications, such as hemorrhage or rupture. Serum β-hCG levels have also been used to diagnose ectopic pregnancy. A β-hCG level >2000 mIU/mL with no signs of intrauterine pregnancy is highly suggestive of ectopic pregnancy [1].

The management of ectopic pregnancy depends on the location and severity of the condition. The goal of management is to preserve fertility and minimize morbidity and mortality rates. In stable patients with an unruptured ectopic pregnancy, β-hCG levels <5000 mIU/mL and no fetal cardiac activity can be managed expectantly or medically using methotrexate (MTX). Methotrexate is a folic acid antagonist that disrupts rapidly dividing cells, including trophoblasts, of the gestational sac. Contraindications to medical management include hemodynamic instability, anemia, leukopenia, thrombocytopenia, pelvic pain, or hemoperitoneum indicative of EP rupture, renal or hepatic insufficiency, pulmonary disease, active peptic ulcer disease, coinciding IUP, breastfeeding, fetal cardiac activity, serum β-hCG levels > 5000 mIU/mL, or ectopic pregnancy > 3.5 cm in diameter [7]. Methotrexate can be administered as a single-dose or a two-dose regimen. The choice of regimen was based on the patient’s β-hCG level. Patients with higher β-hCG levels may benefit from a two-dose regimen than from a single-dose regimen [8].

Surgical intervention may be necessary in more severe cases, such as large ectopic pregnancies (>3.5 cm), viable ectopic pregnancies, β-hCG level >5000 mIU/mL, ruptured ectopic pregnancies, or hemodynamically unstable patients [9]. It can be performed via laparoscopy or laparotomy, depending on the clinical situation. Surgical management aims to remove ectopic pregnancies and minimize blood loss. Salpingectomy and salpingostomy are the two main approaches for surgical treatment of ectopic pregnancies. Salpingectomy is a procedure in which the damaged fallopian tube is completely dissected and removed, whereas salpingostomy is a procedure in which only ectopic pregnancy is removed through a small incision in the fallopian tube [10]. Salpingectomy is recommended in cases where the ectopic pregnancy is >5 cm in diameter [7]. In some cases, diagnostic laparoscopy may be performed if an ectopic pregnancy is not visualized. In rare cases, where ectopic pregnancy occurs in the cervix or broad ligament, conservative management with local injection of methotrexate or selective arterial embolization may be considered [5]. Overall, surgical management has shown high success rates compared with conservative and medical management with methotrexate [7].

Expectant management is considered in patients who are hemodynamically stable with declining or plateauing β-hCG levels [5]. This involves close monitoring of the patient with serial serum β-hCG levels (every 48 hours) and ultrasound scans. In cases of non-declining β-hCG levels, other options should be considered and discussed with the patient [10].

Ectopic pregnancy is a serious medical condition requiring prompt diagnosis and appropriate management. Understanding the causes and risk factors of ectopic pregnancy is essential for early intervention and the prevention of complications. Early diagnosis and prompt management can significantly reduce the maternal morbidity and mortality associated with ectopic pregnancies.

Despite significant improvements in the diagnosis and management of ectopic pregnancy, it remains a significant cause of maternal morbidity and mortality. Further research is needed to better understand the underlying mechanisms of ectopic pregnancy and develop more effective prevention and treatment strategies. Future studies should focus on identifying novel diagnostic biomarkers and developing new medications that can effectively treat ectopic pregnancies without the need for surgical intervention. Additionally, more research is needed to evaluate the long-term outcomes of different management strategies for ectopic pregnancy, particularly in terms of fertility and future pregnancy outcomes.

## Materials and methods

Ethical considerations

All patient records were collected from the electronic health system (SALAMA) at Dubai Academic Health Corporation-Latifa Hospital. All records were reviewed in the inpatient registry. Patients who had been diagnosed with ectopic pregnancy (ICD 10 code O00.8, O00.9) were included in this study. The data accessed was from 1st January 2018 and 31st December 2019. All patients consented. Patient records were fully anonymized prior to access.

The study protocol was reviewed and approved by the Dubai Scientific Research Ethical Committee (DSREC) of Dubai Academic Health Corporation; the ethical approval number of the study is DSREC/RRP/2022/19.

Study design and study setting

This was a retrospective observational study conducted on women who had been diagnosed with ectopic pregnancy at a high-volume tertiary center in Dubai between January 2018 and December 2019. The Medical Record Number (MRN) of the patients and the data were collected from electronic medical records (SALAMA) after filtering the patients who were diagnosed with ectopic pregnancy in the years 2018 and 2019.

Sample size calculation

To determine the appropriate sample size for this cross-sectional study, we utilized a formula that considers the desired confidence level, margin of error, and population size. Dubai's female population is approximately 1,025,100 [11], with a confidence level of 85% and a margin of error of 5%. The sample size was calculated using the formula (n = (Z2 ⋅ p⋅(1-p))/e2). Here, (Z) represents the Z-value corresponding to the 85% confidence level (approximately 1.44), (p) is the estimated proportion of the population (assumed to be 0.5 for maximum variability), and (e) is the margin of error (0.05). Substituting these values into the formula, we obtained a sample size of 208. This ensures that the study results will be statistically significant and representative of the larger population.

Study population and procedures

The study examined all patients of various nationalities diagnosed with ectopic pregnancy at Latifa Hospital from January 1, 2018, to December 31, 2019, who met the inclusion criteria (Table 1); ultimately, 237 women with abnormal pregnancy implantation were identified, although 28 were excluded for not fulfilling these criteria. Collected variables included age (categorized as above or below 30 years), nationality (UAE national vs. non-UAE national), and body mass index (BMI, calculated as weight in kilograms divided by height in meters squared and recorded to one decimal point) was classified according to CDC guidelines as shown in Table 2 [12], and parity (nullipara or multiparous). Miscarriages were defined as spontaneous pregnancy losses before fetal viability (up to 24 weeks gestation) and classified as a dichotomous variable (yes/no) [13]. Beta hCG levels were categorized as <5000 mIU/mL or ≥ 5000 mIU/mL, while hemoglobin (Hb) levels were classified as <9 g/dL or ≥ 9 g/dL upon hospital presentation. The adnexal mass size was measured by ultrasound in width and length; the presence of fetal cardiac activity and the presence of free fluid was graded as minimal, mild, moderate, or severe based on ultrasound findings. Vital signs at presentation were classified as stable, hypotensive, tachycardic, or both hypotensive and tachycardic. Additionally, the history of ectopic pregnancy and abdominal surgery were recorded as dichotomous variables (yes/no), and the use of an intrauterine contraceptive device (IUCD) and the use of assisted reproductive techniques (ART) were similarly classified. Management types were categorized as one dose of methotrexate, two doses of methotrexate, or surgical management, with surgical types further classified, and the need for additional intervention post-initial management was also noted as a yes/no variable. Methotrexate doses were calculated using the following formula for a single-dose regimen: Methotrexate 50 mg/body surface area (m2), with beta hCG level being measured on days 4 and 7. The patient required further management when the beta hCG drop was <15% between days 4 and 7. In contrast, for the two-dose regimen, the dose was calculated by Methotrexate at 1 mg/kg and was given on days 1 and 3 with follow-up of beta-hCG daily. The failure of treatment was considered if the repeated beta hCG levels failed to drop >15% [5].

**Table 1:** Inclusion and exclusion criteria

Inclusion criteria	Exclusion criteria
All females of reproductive age group	Patients who signed refusal of treatment
Diagnosis of tubal (ectopic) pregnancy	Diagnosis of pregnancy of unknown location
Medical management	Diagnosis of scar pregnancy
Surgical management	Expectant management

**Table 2:** CDC categories of BMI ranges for adults

BMI	Weight status
Below 18.5	Underweight
18.5 – 24.9	Normal or healthy weight
25.0 – 29.9	Overweight
30.0 and above	Obese

Data analysis

Statistical analyses were performed using IBM Corp. Released 2011. IBM SPSS Statistics for Windows, Version 20.0. Armonk, NY: IBM Corp. Descriptive statistics were used to summarize the data. Continuous and categorical data were expressed as mean+/-standard deviation. The distribution of categorical variables was examined using Chi-square statistics. The Chi-Square Test was used to measure the strength of association between the type of management and clinical variables. P <0.05 was considered statistically significant.

## Results

Demographic and reproductive health characteristics of women

The demographic characteristics of the study population are shown in Table 3. In total, 209 participants were included in this study.

**Table 3:** Demographic data and obstetrics characteristics of participants (n=209)

Characteristics		N=209	%
Age (mean)		30.3 ± 4.5 years	
Nationality	UAE	78	37.3
	Non-UAE	131	62.7
BMI	Underweight	7	3.3
	Normal weight	70	33.5
	Overweight	76	36.4
	Obese	56	26.8
Parity	Nulliparous	84	40.2
	Multiparous	125	59.8
Miscarriages	Yes	70	33.5
	No	139	66.5
Previous ectopic pregnancy	Yes	25	12
	No	184	88
Use of ART	Yes	3	1.4
	No	206	98.6

The mean ± SD age was 30.2 ± 4.5 years. As shown in Table 3, 111 participants were above or equal to 30 years of age, which represents more than half (53%) of the total participants. Most participants were non-UAE nationals (131 [63%]). Of the 209 participants, 132 (63.2%) had a BMI above the normal range (overweight and obese). Among the total participants, 125 (59.8%) were multiparous, and 84 (40.2%) were in their first pregnancy. Seventy (33.5%) participants had a history of miscarriage. In contrast, 25 (12%) participants had a history of ectopic pregnancy. Adding to that, only 3 (1.4%) participants had a history of ART. 

Factors associated with ectopic pregnancy

**Figure 1:**
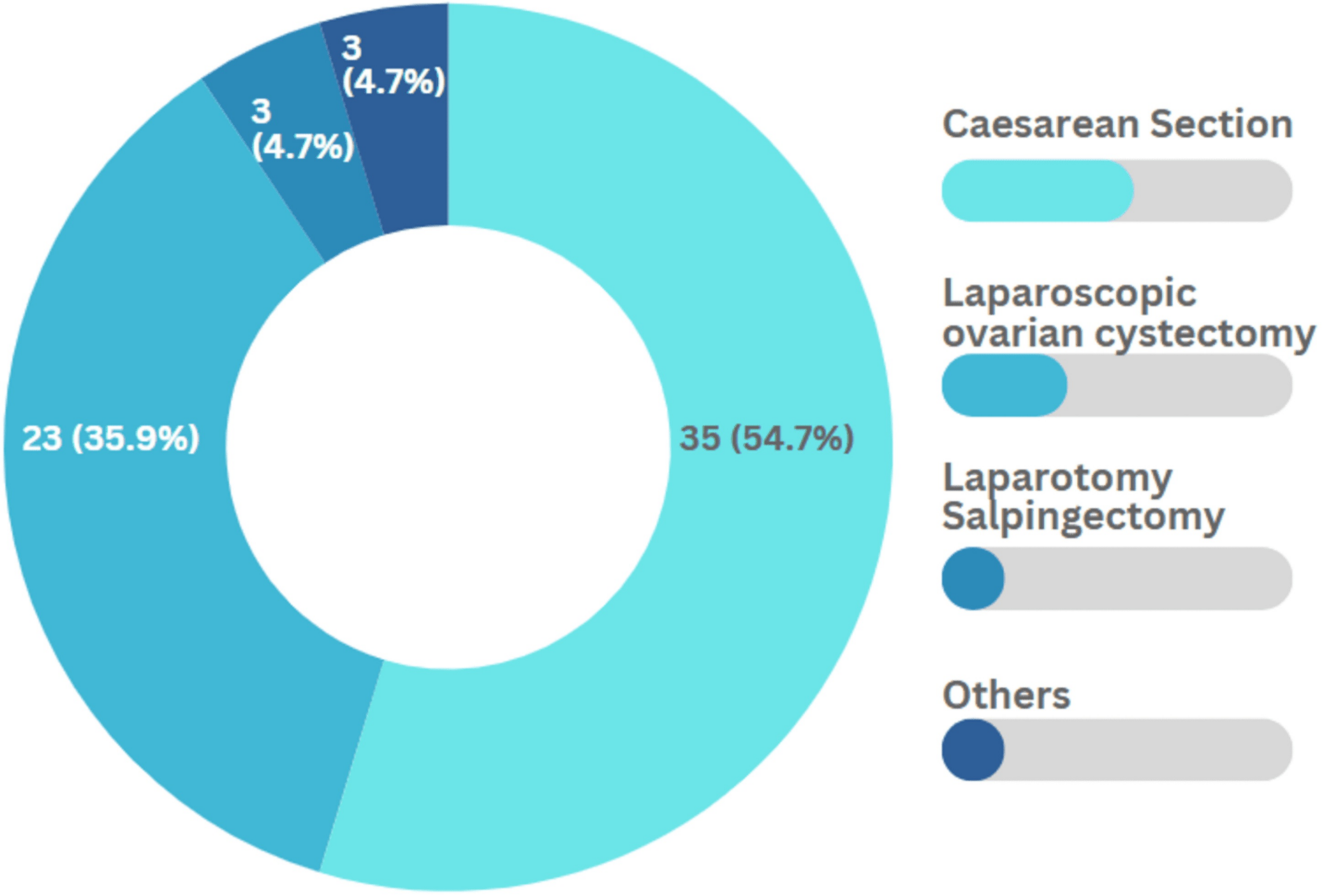
Type of previous abdominal surgery (n=64)

In this study, 64 (30.6%) participants had a history of abdominal surgery. Out of which more than half (35 [54.7%]) had a history of a cesarean section, as shown in Figure 1. Adding to that, six (3%) participants had an intrauterine contraceptive device (IUCD) inserted and became pregnant while the IUCD was still in situ.

Frequency of use of each modality in the management of ectopic pregnancies

**Figure 2:**
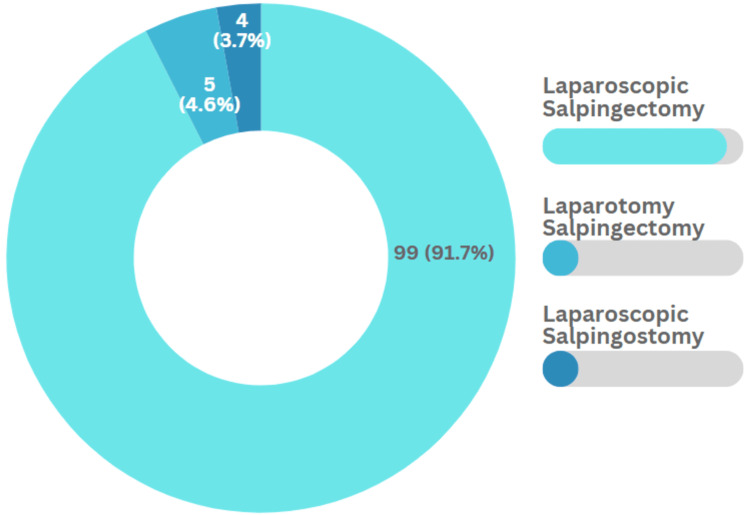
Frequency of each type of surgical management (n=108)

Most of the participants (108 [52%]) underwent surgery. Out of which, the majority (99 [91.7%]) underwent laparoscopic salpingectomy (Figure 2). In contrast, of the 101 participants who underwent medical management by methotrexate injections, 77 (76.3%) received one dose of methotrexate and 24 (23.7%) received two doses of methotrexate.

Clinical indicators associated with the determination of management use

All seven clinical indicators assessed to determine the type of management used showed statistical significance. All patients who were hypotensive and tachycardic underwent surgery. In addition to that, most patients who were tachycardic underwent surgical management. Furthermore, it was found that the majority of patients who had β-hCG levels of more than or equal to 5000 mIU/mL underwent surgical management. Moreover, patients with hemoglobin levels of less than 9 g/dL were more likely to be managed surgically, out of which only nine (8.4%) patients required blood transfusion and were admitted to the ICU for observation (Table 4).

**Table 4:** Crosstabulation between clinical indicators and type of management

Clinical Indicators		One dose of MTX	Two doses of MTX	Surgical management	P-value
		N (%)	N (%)	N (%)	
		77 (37)	24 (11)	108 (52)	
Vital Signs	Stable	71 (92.2)	21 (87.5)	77 (71.3)	0.003
	Hypotensive	5 (6.5)	2 (8.3)	8 (7.4)	
	Tachycardic	1 (1.3)	1 (4.2)	13 (12)	
	Hypotensive and tachycardic	0	0	10 (9.3)	
β-HCG Level	< 5000 mIU/mL	69 (89.6)	20 (83.3)	58 (53.7)	<0.001
	≥ 5000 mIU/mL	8 (10.4)	4 (16.7)	50 (46.3)	
Hemoglobin Level	< 9 g/dL	6 (7.8)	1 (4.2)	28 (25.9)	0.001
	≥ 9 g/dL	71 (92.2)	23 (95.8)	80 (74.1)	
Presence of Free Fluid on Ultrasound	No free fluid	33 (42.9)	11 (45.8)	22 (20.4)	<0.001
	Minimal	7 (9.1)	4 (16.7)	7 (6.5)	
	Mild	28 (36.3)	5 (20.8)	24 (22.2)	
	Moderate	8 (10.4)	3 (12.5)	38 (35.2)	
	Severe	1 (1.3)	1 (4.2)	17 (15.7)	
Mass Size on Ultrasound	No mass	12 (15.6)	4 (16.7)	16 (14.8)	0.001
	<3.5 cm	56 (72.7)	14 (58.3)	50 (46.3)	
	≥ 3.5 cm	9 (11.7)	6 (25)	42 (38.9)	
Presence of Fetal Cardiac Activity	Yes	0	0	25 (23.2)	<0.001
	No	77 (100)	24 (100)	83 (76.8)	
Need for Blood Transfusion	Yes	0	0	9 (8.4)	0.012
	No	77 (100)	24 (100)	99 (91.6)	

Transvaginal ultrasound reports were used for the three clinical indicators (presence of free fluid, mass size, and presence of fetal cardiac activity), and it was found that patients who had findings of moderate to severe free fluid, those who had a mass measuring ≥ 3.5 cm, and those with positive fetal cardiac activity were more likely to be managed surgically (Table 4).

Efficacy of each modality in the management of ectopic pregnancies

As shown in Table 5, of the 209 participants, 177 (84.7%) did not need any further intervention after the initial management. Of the 32 (15.3%) participants who needed further intervention, 19 (59.4%) received one dose of methotrexate as their initial management, 10 (31.2%) received two doses of methotrexate, and three (9.4%) were treated with surgical management by laparoscopic salpingostomy.

**Table 5:** Crosstabulation between management and need for further intervention

Type of management used		One dose of MTX	Two doses of MTX	Surgical management	Total	P-value
		N (%)	N (%)	N (%)	N (%)	
		77 (37)	24 (11)	108 (52)	209 (100)	
Further Treatment Needed	No	58 (75.3)	14 (58.3)	105 (97.2)	177 (84.7)	<0.001
	Yes	19 (24.7)	10 (41.7)	3 (2.8)	32 (15.3)	

## Discussion

The current study was conducted to assess the frequency of medical and surgical management in the treatment of ectopic pregnancy among patients who attended Latifa Hospital and the efficacy of each modality used. According to ACOG, overall ectopic pregnancy is diagnosed in 1-2% of all pregnancies, and it accounts for 5-10% of pregnancy-related deaths worldwide [5]. In the present study, 209 patients were diagnosed with ectopic pregnancy in Latifa Hospital during 2018 and 2019. More than half the patients were above or equal to the age of 30 years. This finding corresponds to the fact that advancing maternal age (above 35 years) increases the risk of having ectopic pregnancy [14]. That is due to different hypotheses, which include increased chromosomal abnormalities in trophoblastic tissue and age-related changes in tubal function delaying ovum transport, resulting in ectopic implantation of the pregnancy [15].

The majority of participants (62.7%) were non-nationals. This result is reflected in the general population, as 91% of Dubai’s population is composed of foreigners [16]. In addition to that, 63.2% of the participants had a BMI above 25, classifying them into overweight and obese categories. In contrast, only 3.3% of participants were in the underweight category. However, this could be due to the high prevalence (16-28.4%) of obesity in the UAE [17]. Furthermore, this was inconsistent with the result of the study conducted by Levin et al., in which only 15.9% (31/195 patients) were obese [18].

According to the current study, the majority of participants (59.8%) who were diagnosed with ectopic pregnancy fell into the multiparous group. Shafquat et al. reached a similar conclusion that showed that ectopic pregnancy was most frequently seen in multiparous women (47.3%) as compared to primiparous (34.66%) and grand multiparous women (18.8%) [19]. Another finding was that 33.5% of participants had a previous history of miscarriage. A study conducted in India by Prasanna et al. found that a history of previous miscarriage was found in 16% of patients included in their study [20]. Another study conducted in Nigeria by Igwegbe et al. found a similar finding to the current study [21]. This study showed that only 12% of participants had a history of ectopic pregnancy. The above finding corresponds with a study conducted by Petrini et al., which also showed that the recurrence rate once a patient has a history of ectopic pregnancy is 10-20% [22].

In the present study, it was found that 30.6% of participants had a previous history of abdominal surgery. Adding to that, 16.7% had a history of at least one cesarean section. A study conducted by Shah et al. found that cesarean delivery was a risk factor in 10.5% of patients [23]. Another study conducted in Nigeria by Igwegbe et al. found that a previous history of abdominopelvic surgery was a risk factor in 29% of patients, out of which 6.5% of patients had a history of a cesarean section [21]. When investigating the risk factors of ectopic pregnancy, it was found that only 3% of participants had a history of IUCD usage, and only 1.4% of participants had a history of ART. A study conducted in a tertiary care center in India by Prasanna et al. found a similar finding [20].

Out of the 209 patients who were included in this study, 53% were managed surgically, 37% received a single dose of methotrexate, and only 11% received two doses of methotrexate. Out of the participants who were managed surgically, the majority underwent laparoscopic salpingectomy (91.7%). In Bahrain, the treatment of choice was laparotomy salpingectomy (84.2%), single-dose methotrexate (5.3%), and methotrexate injection followed by laparotomy salpingectomy (10.5%). On the other hand, in Qatar, it was laparoscopic salpingectomy (77.6%), single-dose methotrexate (19.4%), and methotrexate injection followed by laparoscopic salpingectomy (3%) [24]. According to a study conducted in Nigeria, the majority of patients were managed surgically by laparotomy salpingectomy (75.3%), and medical management was used in only 2.2% of patients [21]. However, their study limitation was that most of the patients included in their study were unstable and required immediate intervention. 

In comparison, most patients (80.9%) who presented to our hospital were hemodynamically stable. In this study, only 4.8% of participants were vitally unstable, all of whom underwent surgical management. There is statistical significance between vital signs and the type of management used (p<0.003). The more the patient is hemodynamically unstable, the more likely she will undergo surgical management. Only nine (4.3%) participants required blood transfusion and ICU admission. However, in a study conducted in Chennai, 27.7% of participants required blood transfusions [25].

The majority of participants who had a β-hcg level of ≥ 5,000 mIU/mL were managed surgically. Furthermore, according to the current study, a patient who had signs of ruptured ectopic pregnancy (presence of moderate to severe free fluid in the pelvis), an adnexal mass size ≥ 3.5 cm, or the presence of fetal cardiac activity on transvaginal ultrasound were more likely to be managed surgically. There is statistical significance between these factors and the type of management used (p<0.001). A similar finding was found in a study conducted by Hendriks et al. in which they recommended surgical management for patients with peritoneal signs or hemodynamically unstable if β-hcg level was more than or equal to 5,000 mIU/mL or if there was viable ectopic pregnancy [10].

In this study, the majority of the patients had a hemoglobin level of ≥9 g/dL. However, among participants who had hemoglobin <9 g/dL, the majority underwent surgical management. There is a statistical significance between the level of hemoglobin and the type of management used (p=0.001). According to a study conducted in India by Shraddha Shetty, anemia was found in 41.9% of patients who were diagnosed with ectopic pregnancy [26].

A meta-analysis of non-randomized studies conducted by Barnhart et al. showed that a multidose regimen was more effective than a single-dose regimen [27]. However, the opposite was found in this study, in which a single dose of methotrexate was more effective compared to a two-dose regimen. This could be due to the participant’s choice to preserve fertility by avoiding surgical management. This result is statistically significant (p<0.001).

Study limitations

This study encountered several limitations. Firstly, among the group of patients who received two doses of methotrexate, some were initially indicated for surgical management but opted against it to preserve their fertility. This decision may have influenced the observed efficacy of the treatment. Additionally, a few patients did not adhere to follow-up appointments, either due to financial constraints or because they had relocated. This lack of follow-up potentially impacted the assessment of the efficacy rates and the final outcomes of both interventions.

## Conclusions

In conclusion, this study provides a comprehensive analysis of the demographic and clinical characteristics influencing treatment outcomes in a diverse population. The findings highlight the significant role of age, nationality, BMI, and reproductive history in determining the management strategies for patients. The study underscores the effectiveness of both surgical and medical management protocols, with a majority of participants not requiring further intervention after initial treatment. The clinical indicators, including vital signs, β-hCG levels, hemoglobin levels, and transvaginal ultrasound findings, were pivotal in guiding the choice of management. These indicators demonstrated significant correlations with the likelihood of surgical intervention, emphasizing the importance of thorough clinical assessment in treatment planning.

Despite the study’s limitations, such as patient follow-up challenges and the influence of fertility preservation decisions, the results offer valuable insights into optimizing treatment protocols. The necessity for careful monitoring and follow-up is evident, particularly for patients with complex clinical presentations. Further studies are needed to identify the pathophysiology and molecular causes of ectopic pregnancy to develop preventive measures to avoid recurrence and disease occurrence. Overall, this study contributes to the existing body of knowledge by elucidating the factors that impact treatment efficacy and outcomes. It provides a foundation for future research and clinical practice improvements, aiming to enhance patient care and management in similar populations.
